# Emotional Speech Perception Unfolding in Time: The Role of the Basal Ganglia

**DOI:** 10.1371/journal.pone.0017694

**Published:** 2011-03-17

**Authors:** Silke Paulmann, Derek V. M. Ott, Sonja A. Kotz

**Affiliations:** 1 Department of Psychology, University of Essex, Colchester, United Kingdom; 2 Max Planck Institute for Human Cognitive and Brain Sciences, Leipzig, Germany; Duke University, United States of America

## Abstract

The basal ganglia (BG) have repeatedly been linked to emotional speech processing in studies involving patients with neurodegenerative and structural changes of the BG. However, the majority of previous studies did not consider that (i) emotional speech processing entails multiple processing steps, and the possibility that (ii) the BG may engage in one rather than the other of these processing steps. In the present study we investigate three different stages of emotional speech processing (emotional salience detection, meaning-related processing, and identification) in the same patient group to verify whether lesions to the BG affect these stages in a qualitatively different manner. Specifically, we explore early implicit emotional speech processing (probe verification) in an ERP experiment followed by an explicit behavioral emotional recognition task. In both experiments, participants listened to emotional sentences expressing one of four emotions (anger, fear, disgust, happiness) or neutral sentences. In line with previous evidence patients and healthy controls show differentiation of emotional and neutral sentences in the P200 component (emotional salience detection) and a following negative-going brain wave (meaning-related processing). However, the behavioral recognition (identification stage) of emotional sentences was impaired in BG patients, but not in healthy controls. The current data provide further support that the BG are involved in late, explicit rather than early emotional speech processing stages.

## Introduction

Accumulating neuroanatomical, neuroimaging, neuropsychological, and behavioral evidence has informed our present understanding of emotional speech processing. It has become evident that emotional speech processing is a highly complex endeavor mediated by a differentiated network of fronto-temporal, fronto-parietal cortices, the amygdala, and the basal ganglia (BG). Specifically, it has been proposed that emotional speech processing can be divided into several sub-processes after the first encounter with an emotionally marked stimulus (identification of emotional significance, detailed emotional perception of stimulus, conceptual evaluation; see e.g. [1], [2]). However, while there is growing evidence on the brain structures involved in emotional speech processing, the temporal dynamics of specific sub-processes and their impact on specific brain structures are less clear. For example, imaging studies using PET, fMRI, or TMS do not allow to clearly specify the time-course of activation patterns. However, event-related brain potential (ERP) lesion studies offer the possibility to explore which brain areas are linked to specific processing steps due to their fine grained temporal resolution. Thus, the present study applied ERPs to investigate sub-processes of emotional speech processing in patients with BG lesions in comparison to healthy controls. This patient group was of interest for two reasons: 1) the BG have long been implicated in emotional speech processing [3]–[6], and 2) we previously explored two sub-processes of emotional speech processing (prosody alone and together with semantics) in the same patient group [7], [8], thus results can be directly compared across studies.

### Emotional Speech Processing Steps

When listening to a sentence such as “She won the lottery”, interpretation of the utterance will depend on how the words are intoned (emotional prosody) by the speaker, i.e. we are usually very accurate at identifying whether the speaker is happy about this event or whether they envy the winner. Identifying what and how something is said requires comparing the semantic meaning and the prosody of an unfolding sentence. Recent electrophysiological evidence suggests that the comprehension of emotional prosody and emotional semantics proceeds along two distinct but probably highly interactive and possibly interdependent processing streams which are likely to be subserved by partially overlapping neural networks [9]–[11] (and see [12] for review on participating brain structures). In particular, it is suggested that we 1) need to compute emotional features (e.g. emotional prosodic cue patterns, arousal, valence, emotional meaning) and may 2) establish emotional memory based relationships (i.e. relate emotional connotation of sentence to emotional information stored in memory) resulting in 3) a final sentence interpretation during listening. There is ample evidence from both neuroimaging and electrophysiological studies [10], [13]–[17] that supports the assumption of different emotional speech processing steps. For instance, recent ERP investigations linked the processing of certain acoustic cues such as frequency and intensity information to the so-called N100, an early ERP component that does not respond to the emotional impact of a stimulus [13]. Following this early sensory analysis, the integration of significant emotional acoustical cues and the detection of emotional salience takes place within 200 ms after stimulus onset as, for example, reflected in variations of the P200 amplitude for basic vocal emotions compared to a neutral baseline [14], or in Mismatch Negativity (MMN) differences between emotional and neutral stimuli [15]. In addition, we suggested that not only emotionally relevant details encoded in an auditory stimulus, such as valence [14] or arousal [16] can be inferred within the first 200 ms of stimulus onset, but possibly also emotional category-related knowledge [17]. Finally, later stages of processing have been linked to later negativities such as the N300 and N400, components that have been argued to indicate emotional meaning and integration processes [17]–[22]. Taken together, there is a substantial literature supporting the idea that emotional speech processing can be subdivided into several processing steps that ultimately lead to emotional sentence interpretation.

### The role of the BG during different emotional speech processing steps

For years, the BG [23] have been linked to emotional speech processing [3]–[6], [24]–[26]. In particular, it has been shown that BG impairment often leads to difficulties in recognizing emotions from speech [4], [7], [24], [27]–[29], [30]–[32]. Despite the wealth of evidence gathered over the past years, the specific functional role of the BG in emotional speech processing still awaits further specification. That is, what role do the BG play during the different processing steps outlined above? Applying ERP-lesion studies can help specifying the functional role of the BG during different emotional processing steps.

For instance, we examined emotional prosodic deviance detection in patients with focal lesions in the left BG during implicit on-line emotional processing (probe verification task). In addition, the same patient group was tested during explicit processing of emotional prosody in an off-line emotional prosody recognition task [7]. ERP components in response to emotional prosodic expectancy violations were comparable between BG patients and healthy participants; however, patients were significantly impaired in the explicit judgments of emotional prosody when compared to healthy controls. These results suggest that the left BG may not play a mandatory role during *implicit* processing of emotional prosody but that processes implied during explicit emotional prosody recognition or categorization tasks would be modulated more strongly by the BG.

One question that naturally follows from these results relates to the fact that on-line and off-line processing was tested with two different task instructions (probe detection vs. emotional prosody categorization). Thus, the discrepancy could be task- related and not necessarily due to differential BG involvement in different processing steps (e.g. early vs. late). In fact, it has previously been argued that the BG specifically engage in *executive* processes, suggesting a role for the BG in the explicit evaluation (recognition/categorization) of vocal emotion expressions [33]. For instance, Bach and colleagues conducted an fMRI study that investigated emotional prosody processing with implicit (gender labeling) and explicit (emotion labeling) task instructions. The authors reported stronger BG involvement for emotional vs. neutral prosody processing when participants labeled emotions of stimuli, implying a prominent role for the BG during explicit task instructions [33].

The specific functional role of the BG with explicit task instructions has also been addressed: one possible role for the BG could be related to sequencing and binding auditory (emotional) information [4], [34]. In particular, it has been proposed that the BG are part of “integrational processes which occur at a late stage during sentence comprehension” [34]. This hypothesis has received support from both emotional [8] and non-emotional language [25], [35], [36] investigations that report BG involvement during “late” evaluative, integration, and recognition related responses, but no such involvement in early, more automatic processing stages (but see [37] for rare evidence on impaired early sensory processing in PD patients). For instance, recent data [8] confirm that BG impairment can lead to a deregulated emotional cue integration process. Specifically, the on-line integration of emotional semantic and prosodic features was studied by recording ERPs in response to combined emotional prosodic and semantic expectancy violations (i.e. a detection of abrupt semantic content and speaker tone change). Results revealed an altered capacity to combine information from the two sources (prosody & semantics) in BG patients when compared to healthy controls [8]. Interestingly, this impairment was found under *implicit* task instructions, rendering it unlikely that dissociations between ERPs and behavioral results as reported in [7] were only due to differences in task instructions/focus but instead highlight the possibility that the BG may be involved in functionally different processing steps. Thus, we hypothesized that the BG may be crucial for binding emotional cue relations especially in tasks or processes which *enforce* an integrative *evaluation* of emotional information [7], [8]. This means that the BG potentially play a role during early *and* late stages of emotional speech processing, but this involvement should depend on task demands and stimulus-type manipulations [8].

### The present investigation

Building on the results summarized above, the present study aimed to further test in which way the BG engage during early and late emotional speech processing steps under implicit and explicit task instructions. Specifically, we explore the sub-processes of emotional salience detection (P200), combining incoming information into an emerging emotional representation (N300/N400), as well as decision-making stages (as indicated by behavioral results) in patients with left BG impairment and healthy controls. To this aim, patients were tested in two different experiments: 1) the ERP experiment tested early and late stages using an implicit emotional task instruction (probe verification), i.e. task instructions/goals do not emphasize the emotional nature of sentences. Here, both amplitude and latency measures were scrutinized as they can inform about processes involved in emotional speech perception. In particular, studies with healthy participants have shown that neutral sentences can be differentiated from emotional sentences in the P200 amplitude [14]. Furthermore, it has been suggested that this early emotional salience detection is crucial for further processing steps, especially if emotional stimuli are to be prioritized [38]. Such prioritization could be reflected in a temporal lag comparing emotional and neutral sentences and affect subsequent processing steps reflected in later ERP components. We expect to find differences between healthy controls and BG patients if the BG are implicated in one or both of these sub-processes (salience detection, building-up of emotional meaning representation). 2) The behavioral experiment tested explicit emotional speech identification accuracy of vocal expressions such as anger, fear, disgust and happiness compared to a neutral baseline. If the BG are involved primarily in evaluative judgment (executive) functions this should be reflected in impaired behavioral responses of BG patients when compared to healthy controls.

## Methods

### Ethics Statement

All participants gave informed written consent before completing the study, which was ethically approved by the Max Planck Institute for Human Cognitive and Brain Sciences Review Board.

### Participants

**Figure 1:**
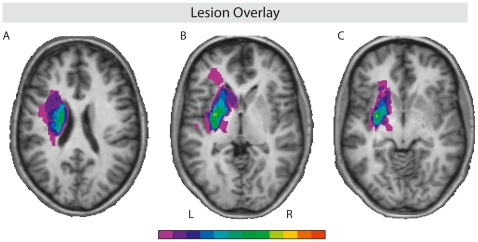
Lesion overlay. This illustration shows an overlay of the respective individual patient lesions indicating maximum overlap in the basal ganglia. Display A: Talairach coordinates (x,y,z): −29, −5, −2. Left corpus nuclei caudati (lesions extend to white matter adjacent to the lateral ventricular wall and inferior frontal, central and precentral sulci). Display B: Talairach coordinates (x,y,z): −28, −3, −4. Left middle-posterior putamen and globus pallidus (lesions extend to the head of the caudate nucleus; internal, external and extreme capsule; posterior insula and deep frontal with matter). Display C: Talairach coordinates (x,y,z): −17, −5, −23. Left inferior middle-posterior putamen (lesions extend to ventromedial striatum). Green/yellowish shades reveal maximum overlap of lesion sites, whereas purple shades reveal minimal lesion site overlap.

Twelve native speakers of German (1 female, all right-handed; mean age: 49.2 years, SD: 17.2) with focal lesions in the striatum participated in the study. Brain lesions of participants resulted from LH insults: ischemic stroke (n = 3), embolic stroke (n = 2), intracerebral bleeding (ICB; n = 6), or arterio-arterial infarction (n = 1). The average time post-lesion was: 4.6 years (range 1.8–7.1). Lesion sites were determined by (T1- and T2-weighted) anatomical MRI datasets from a 3.0 T system (Bruker 30/100 Medspec) and evaluated by an experienced neuroanatomist. All patients were non-aphasic. Individual patient information are reported in Table 1, neuropsychological test results in Table 2. In addition, twelve healthy controls, matched for age, gender, and education, were tested. See Figure 1 for a graphical display of a lesion overlay.

**Table 1:** Demographic information.

Patient	Time since lesion (years)	Age at test (years)	Sex	Etiology	Lesion description
01	7 yrs 4 mos	63	m	ICB	ant. GPe, ant. IC
02	6 yrs 1 mos	53	m	ICB	post. Put., GPe, post. EC, IC, lat. Thal.
03	5 yrs 1 mos	48	m	ICB	Put., GPe, EC, ant. IC, reduced volume of Caud.
04	5 years 5 mos	31	m	Ischemic Infarction	post. Put., Caud. (body), middle Ins., parietal operculum
05	4 yrs 4 mos	68	m	Ischemic Infarction	Caud. (ant. body), ant. Put., GPe, EC, ant. IC, ant. Ins., preinsular WM
06	3 yrs 3 mos	40	f	Arterio-Arterial Infarction	Caud. (body), Put., GPe, ant. IC, EC, parietal operculum, post. Ins.
07	4 yrs 11 mos	59	m	Ischemic Infarction	Caud. (body), Put., GPe, IC, EC
08	7 yrs 11 mos	66	m	ICB	Caud., Put.
09	6 yrs	33	m	Embolic Infarction	Put., Caud.
10	1 yrs 8 mos	28	m	ICB	post. Put., Caud.
11	3 yrs 5 mos	26	m	ICB	Thal., post. Put., Caud.
12	4 yrs 11 mos	75	m	Embolic Infarction	Caud. (body), Put.,

Lesions resulted from left hemispheric insults. The average time since lesion in the BG was: 4 years and 6 months. Lesion sites were determined by (T1- and T2-weighted) anatomical MRI datasets from a 3.0 T system (Bruker 30/100 Medspec) and evaluated by an experienced neuroanatomist. Abbreviations: m: male; f: female; ICB: intracerebral bleeding; ant: anterior; post: posterior; Caud: caudate nucleus; EC:, external capsule system; IC: internal capsule; Ins: insula; Gpe: globus pallidus externus; Gpi: globus pallidus internus; Put: Putamen; Thal: thalamus; WM, white matter.

**Table 2:** Neuropsychological Test Results.

Patient	DS1	DS2	TAP1	TAP2	TAP3
1	48	12	18	16	10
2	<2	<2	62	46	2
3	35	5	31	38	84
4	12	5	34	31	16
5	20	2	69	90	58
6	75	5	38	24	34
7	3	12	18	54	5
8	NA	NA	NA	NA	NA
9	12	17	16	14	5
10	97	>98	8	8	3
11	NA	NA	NA	NA	NA
12	76	53	92	54	16

Mean results from BG patients on standardized neuropsychological testing (TAP: Test Battery for Attentional Performance: [67]). Note: DS1 (digit span forward), DS2 (backward), TAP1 (tonic alertness), TAP2 (phasic alertness), TAP3 (divided alertness). NA =  not available.

### Stimulus Material

The stimulus material consisted of semantically and prosodically matching stimuli conveying one of four emotions (*angry*, *disgust*, *fear*, *happiness*) or *neutral* affect. Sentences were spoken by a trained male speaker, and were taped with a video camcorder (SONY Digital Video camera Recorder MiniDV DCR-TRV60E) attached to a high-quality clip-on microphone. The video-material was digitized, and the voice-track was separated from the visual-track. In the current experiment, only voice material was tested. The voice material was digitized at a 16-bit/44.1 kHz sampling rate, and the amplitudes were individually normalized (with *CoolEdit* Version 2000). The stimulus material was prosodically analyzed (see Table 3).

**Table 3:** Acoustic Analyses.

	Sentence Onset to Noun Onset									
Emotion	*duration* *(sec)*	*F0* *(Hz)*	*range* *F0* *(Hz)*	*intensity* *(dB)*	*range intensity* *(dB)*					
ANGER	2.54	256.88	211.09	72.85	56.75					
	0.26	24.91	31.79	2.01	4.45					
DISGUST	2.45	130.86	193.79	69.01	43.86					
	0.24	24.14	105.48	2.67	3.15					
FEAR	3.86	125.32	178.76	68.52	41.78					
	1.22	11.73	100.33	3.75	3.42					
HAPPY	2.41	141.00	165.87	69.02	46.63					
	0.25	14.28	70.31	2.96	3.75					
NEUTRAL	2.43	126.74	189.73	70.65	42.35					
	0.21	9.60	89.53	3.57	4.42					

	Sentence Onset to Noun Onset					Noun Onset to Verb 1 Onset				
Emotion	*duration* *(sec)*	*F0* *(Hz)*	*range* *F0* *(Hz)*	*intensity* *(dB)*	*range intensity* *(dB)*	*duration* *(sec)*	*F0* *(Hz)*	*range* *F0* *(Hz)*	*intensity* *(dB)*	*range intensity* *(dB)*
ANGER	0.49	233.81	111.73	73.74	46.59	0.51	271.08	113.59	74.98	38.79
	0.06	23.66	25.15	1.79	3.77	0.16	30.48	43.67	4.57	18.42
DISGUST	0.48	118.54	49.11	70.85	37.47	0.44	147.68	68.66	73.50	28.97
	0.06	20.85	68.80	2.85	4.43	0.14	42.74	66.91	3.89	11.98
FEAR	0.87	119.19	54.55	68.92	37.52	0.86	128.22	77.44	69.76	33.63
	0.42	11.85	50.93	4.36	3.42	0.50	14.94	85.32	6.12	8.94
HAPPY	0.44	116.78	43.46	70.89	40.01	0.48	160.45	102.48	70.70	35.30
	0.06	16.06	61.86	2.19	4.44	0.12	28.26	64.84	5.27	12.86
NEUTRAL	0.45	120.43	40.88	73.64	35.40	0.42	124.41	38.42	73.47	27.45
	0.07	10.02	54.51	4.05	7.75	0.12	15.98	51.65	4.87	12.44

	Verb 1 Onset to Verb 2 Onset					Verb 2 Onset to Sentence Offset				
Emotion	*duration* *(sec)*	*F0* *(Hz)*	*range* *F0* *(Hz)*	*intensity* *(dB)*	*range intensity* *(dB)*	*duration* *(sec)*	*F0* *(Hz)*	*range* *F0* *(Hz)*	*intensity* *(dB)*	*range intensity* *(dB)*
ANGER	0.70	267.55	132.99	74.41	42.30	0.84	251.94	199.59	71.11	51.43
	0.11	26.57	31.66	3.12	12.61	0.14	33.08	36.14	3.96	12.64
DISGUST	0.72	129.23	125.22	69.65	37.53	0.81	137.79	122.59	65.62	40.94
	0.12	22.33	106.94	3.65	7.69	0.10	37.26	105.81	3.99	4.30
FEAR	1.23	124.73	81.35	68.16	39.56	0.90	128.34	110.28	69.69	36.25
	0.52	16.47	74.92	4.64	3.79	0.23	12.04	101.56	4.36	7.83
HAPPY	0.84	144.94	105.19	67.79	43.23	0.65	137.30	100.41	68.54	41.29
	0.15	27.57	78.69	5.08	6.10	0.08	24.00	67.61	4.57	5.47
NEUTRAL	0.81	137.09	144.03	70.00	38.33	0.74	118.54	100.94	68.17	38.80
	0.14	17.87	86.39	4.23	6.71	0.10	20.52	94.96	4.67	5.62

The Table shows results of the acoustical analyses of sentences. Measurements are calculated from sentence onset to sentence offset (top), as well as from sentence onset to noun onset (left middle), noun onset to first verb onset (right middle), first verb onset to second verb onset (left bottom), and second verb offset to sentence offset (right bottom). Means for different measurements (duration, pitch, intensity) are listed in the upper part of a row and respective standard deviations in the lower part of a row.

Words in sentences were controlled for letter and syllable length, initial sounds, and plosive consonants. In addition, the noun and verb were controlled for word frequency. Table 4 lists example sentences.

**Table 4:** Example Sentences.

Emotion	Example Sentence
ANGER	Er hat das Paar gereizt und aufgebracht.(*He has teased and upset the couple*.)
DISGUST	Er hat die Müllhalde bewohnt und gestunken.(*He has lived in the dump and stunk*.)
FEAR	Er hat die Spuren verwischt und verschleiert.(*He has blurred and disguised his traces*.)
HAPPINESS	Sie hat die Trauung verkündet und gelächelt.(*She has announced the wedding and smiled*.)
NEUTRAL	Sie hat den Eimer geleert und weggelegt.(*She has emptied and put away the bucket*.)

The table lists German example sentences. English literal translations are provided in brackets.

#### ERP experiment

In the ERP experiment, 30 sentences in each emotional category were presented, resulting in a total of 150 lexical sentences. Incidental to this report, an equal amount of pseudo-sentences (sentences without semantic content) and 240 cross-spliced sentences were also presented (see [7], [8] for further details and results).

#### Behavioral Recognition Experiment

The ERP study was followed by a classical forced-choice emotional prosody recognition study. Here, a subset of sentences (10 from each emotional category and neutral for both lexical- and pseudo-sentences) were presented, resulting in 100 trials (see [7] for pseudo-sentences results). The emotional category for each sentence was obtained in an earlier rating study [39]. In this study, 64 participants (32 female) rated the sentences according to their emotion (forced-choice task) and emotional intensity. The sentences presented in the current study were the top-10 highest rated from the previous rating study, hence ensuring very good quality of emotional portrayal (with mean recognition rate obtained from healthy participants ranging above 80% correct).

### Procedure

#### ERP experiment

Participants were seated in a comfortable chair at a distance of 115 cm from a computer monitor. Each participant was tested individually in an electrically shielded room with a two-button panel placed before him/her. Half of the participants pressed the yes-button with their right hand and the no-button with their left hand. The other half proceeded vice versa. Stimulus material was presented via loudspeaker at a comfortable loudness level. Participants were asked to listen to each sentence, to read the following word (flashed with 0 ms delay after sentence offset on the screen for 300 ms), and to make a decision on the probe as quickly and accurately as possible (i.e. participants had to decide whether the probe had occurred in the previously heard sentence). Distribution of probe words was counterbalanced across the experiment. Participants had to respond within 8000 ms. The inter-trial interval was 1500 ms. Before the actual experiment, a practice session with 20 trials was carried out. The main part of the EEG experiment had a run-time duration of approx. 60 minutes (note that individual experiment length may have varied as participants were able to self-determine the length of breaks between blocks).

#### ERP Oddball Experiment

To ensure that potential differences between BG patients and healthy controls were not due to a more general attentional deficit in patients, a P300 oddball paradigm was conducted before the start of the actual ERP experiment. In this experiment participants heard standard tones (600 Hz) with a probability of .8 and deviant tones (660 Hz) with a probability of .2. A total of 500 stimuli were presented. All stimuli lasted for 200 ms and were presented with a constant inter-stimulus interval of 600 ms. The run-time duration of this experiment was seven minutes.

#### Behavioral Recognition Experiment

The behavioral emotional recognition study was carried out after the ERP experiment in the same sound-attenuating booth. All participants had at least 25 minutes break time between the ERP experiment and the behavioral study. Each participant was tested individually, and was seated comfortably with a five-button panel placed before him/her. Each response button on the response panel was labeled with a name of one of the emotional categories tested. Stimulus material was presented via loudspeaker. Participants were directed to listen to the presented sentence and to make a decision as accurately as possible, which emotional category the emotional prosody of the presented sentences corresponded to. Answers had to be given within 8000 ms. The inter-trial interval was 1500 ms. A practice session preceded the experiment. The total run-time duration of the behavioral experiment was approx. 10 minutes.

### ERP Recording

The electroencephalogram (EEG) was recorded from 32 Ag-AgCl electrodes mounted in an elastic cap (Electro-Cap International) according to the modified expanded 10–20 system [40]. Bipolar horizontal and vertical EOGs were recorded for artifact rejection purposes. Signals were recorded continuously with a band pass between DC and 70 Hz and digitized at a sampling rate of 250 Hz. Electrode resistance was kept below 5 K-Ω. The reference electrode was placed on the tip of the nose. Data was re-referenced offline to linked mastoids. Eye artifact control measures were applied to the raw data of each participant to increase the number of critical trials in each [41]. Subsequently, individual EEG recordings were scanned for additional artifacts on the basis of visual inspection. ERPs were filtered off-line with a digital FIR bandpass filter ranging from 0.298 to 30 Hz (−6 dB cutoff; 1471 points). ERPs were averaged for epochs of 800 ms starting 200 ms before sentence onset thus including a 200 ms pre-stimulus baseline. Based on previous findings [42] and close visual inspection time windows were defined for further ERP analyses of mean amplitudes. For graphical display only, ERPs were filtered off-line with a 7 Hz low pass filter.

## Results

ANOVAs with *Group* (BG patients/healthy controls) as between-subject factor and the within-subjects factor *Emotion* (anger, disgust, fear, happiness, neutral) were applied. For ERP analyses, the within-factor scalp regions of interest, *ROI*, was included. Each *ROI* defined a critical region of scalp sites: left frontal (LF), F7 F3 FT7; right frontal (RF), F8 FT8 F4; left central (LC), T7 C3 CP5; right central (RC), T8 C4 CP6; left parietal (LP), P7 P3 O1; right parietal (RP), P4 P8 O2; and midline (ML), FZ CZ PZ. The null hypothesis was rejected for p-values smaller than 0.05. The Greenhouse-Geisser correction was applied to all repeated measures with greater than one degree of freedom in the numerator [43]. If post-hoc comparisons exceeded the degrees of freedom, p-values of post-hoc single comparisons were corrected using a modified Bonferroni procedure [44]. Based on previous work [7], we only follow-up contrasts between neutral and emotional sentences in our planned comparisons. Only significant results are reported.

For the P300 oddball, statistical analyses followed the same design as described above but included the within-subjects factor *Probability* (standard vs. deviant) instead of *Emotion*.

Note, that the probe verification task was solely administered to ensure that participants listened attentively to the sentences (overall comprehension of the sentences was good >86%). Thus, results were not further statistically analyzed (see [7], [8] for same procedure).

### Behavioral Results

**Figure 2:**
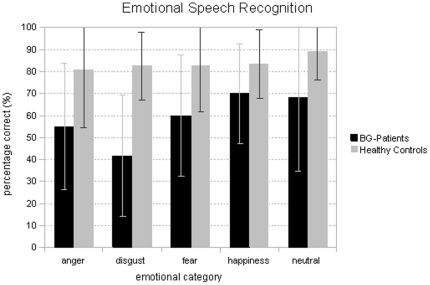
Emotional Speech Recognition. The figure shows mean percentage correct values (incl. standard deviation bars) for each emotional category for both groups for the emotional speech recognition task.

In general, emotional speech recognition was above chance level (20%), for both BG-patients (59%) and healthy controls (84%). Overall, controls showed higher emotional recognition rates than patients. Figure 2 shows mean recognition rates for each emotional category and each group.

The ANOVA of accuracy data confirmed a main effect of *Group* (F(1, 22) = 13.12, p<.01) confirming better emotional prosody recognition rates for healthy controls than for patients. In addition, the *Emotion* effect was significant, (F(1, 22) = 2.90, p<.05). Step-down analyses revealed that neutral sentences (79%) were significantly better recognized than disgust (71%) sentences. No other effects reached significance.

Taken together, the results reveal that emotional speech recognition is worse in BG patients than in matched healthy controls irrespective of emotional category. Table 5 illustrates error distribution for both groups.

**Table 5:** Error distribution.

			Emotion					
	Intended Emotion		*Anger*	*Disgust*	*Fear*	*Happiness*	*Neutral*	*No hit*
**Healthy Controls**	ANGER	*frequency*	-	1	3	0	1	18
		*%*	-	1	3	0	1	15
	DISGUST	*frequency*	2	-	1	4	4	10
		*%*	2	-	1	3	3	8
	FEAR	*frequency*	7	0	-	0	5	9
		*%*	6	0	-	0	4	8
	HAPPINESS	*frequency*	0	0	0	-	9	11
		*%*	0	0	0	-	8	9
	NEUTRAL	*frequency*	0	0	2	0	-	11
		%	0	0	2	0	-	9
**BG-Patients**	ANGER	*frequency*	-	0	14	2	5	33
		*%*	-	0	12	2	4	28
	DISGUST	*frequency*	4	-	7	4	15	40
		*%*	3	-	6	3	13	33
	FEAR	*frequency*	9	6	-	0	8	25
		*%*	8	5	-	0	7	21
	HAPPINESS	*frequency*	0	0	0	-	16	20
		*%*	0	0	0	-	13	17
	NEUTRAL	*frequency*	0	0	1	6	-	31
		%	0	0	1	5	-	26

The table shows the error distribution (frequency and %) as well as no hits (no button press recorded in time-interval) in the behavioral experiment for both groups.

### ERP Results

For the critical main experiment, the ERP component of interest was determined based on previous results [42], mean peak latency and close visual inspection. The time window to calculate ERPs' mean amplitudes was thus set between 180 ms and 280 ms (P200 component) and between 280 ms and 480 ms (following negativity). In addition, a peak-to-peak latency analysis was conducted [45]. To this end, a time-window from 180 ms to 480 ms was set and the mean peak latency from maximum amplitudes (P200 peak) was subtracted from minimum peak amplitudes (negativity). The time window for the classical P300 oddball was set between 200 and 600 ms.

#### ERP oddball experiment

P300 component. Statistical analyses of repeated-measures ANOVA on the P300 effect revealed no significant differences of *Group*, (F(1, 22) = 0.65, p = .4285), but a main effect of *probability* (F(1, 22) = 33.30, p<.0001) indicating that patients and healthy controls both showed a P300 effect.

#### P200 mean amplitudes

Within the time window of 180–280 ms a trend towards a main effect of *Group* (F(1,22) = 3.43, p = .08) was found (with patients showing stronger P200 amplitudes than healthy controls), but no interaction with the factor group was significant. However, a significant main effect of *Emotion* (F(4, 88) = 9.57, p<.0001) was found, indicating waveform differences between different emotional sentences. Breakdown comparisons revealed that neutral sentences differed significantly from disgust (F(1, 22) = 10.37, p<.01), fearful (F(1, 22) = 37.54, p<.0001), and happy (F(1, 22) = 13.78, p = .001) sentences. Contrasts between angry and neutral sentences failed to reach significance but showed a trend into the same direction (F(1, 22) = 3.18, p = .09). For all comparisons, amplitudes for neutral sentences were more positive-going than amplitudes for emotional sentences, showing early emotional and neutral differentiation.

An anonymous reviewer pointed out that it may be helpful if each group was followed-up to confirm that patients show an *Emotion* main effect according to our hypothesis. Despite the missing interaction between the factors *Emotion* and *Group*, we carried out these analyses: results confirm that both groups show a (marginally) significant *Emotion* effect (controls: (F(4, 44) = 2.43, p = .07); patients: (F(4,44) = 9.50, p<.0001).

#### Negativity mean amplitudes

Within the time window of 280–480 ms, again there was only a trend towards a main effect of *Group* (F(1,22) = 3.66, p = .07) once more reflecting general amplitude differences between patients and controls. No interactions with the factor *Group* reached significance. A marginally significant main effect of *Emotion* (F(4, 88) = 2.67, p = .057) was found, indicating waveform differences between the different sentences. Planned post-hoc comparisons revealed that neutral sentences differed significantly from disgust (F(1, 22) = 13.97, p<.01), and fearful sentences (F(1, 22) = 4.77, p<.05). Contrasts between neutral and happy, or neutral and angry sentences were not significant (p>.1). For all comparisons, amplitudes for neutral sentences were less negative-going than amplitudes for emotional sentences, reflecting processing differences between neutral and emotional sentences.

#### Peak-to-peak analysis

In this analysis, no main effect reached significance; however, an interaction between *ROI* and *Emotion* was found, (F(24, 528) = 2.17, p<.05); indicating latency differences between sentences dependent on electrode location. Follow-up comparisons revealed shorter peak-to-peak latencies for fearful in contrast to neutral sentences (F(1, 22) = 6.20, p<.05) at left frontal and right central (F(1, 22) = 21.38, p<.0001) electrode sites. In addition, comparisons revealed shorter peak-to-peak latencies for angry (F(1, 22) = 9.34, p<.001), fearful (F(1, 22)  = 14.58, p<.001), and happy (F(1, 22) = 9.76, p<.001) in contrast to neutral sentences at right frontal electrode sites.

**Figure 3:**
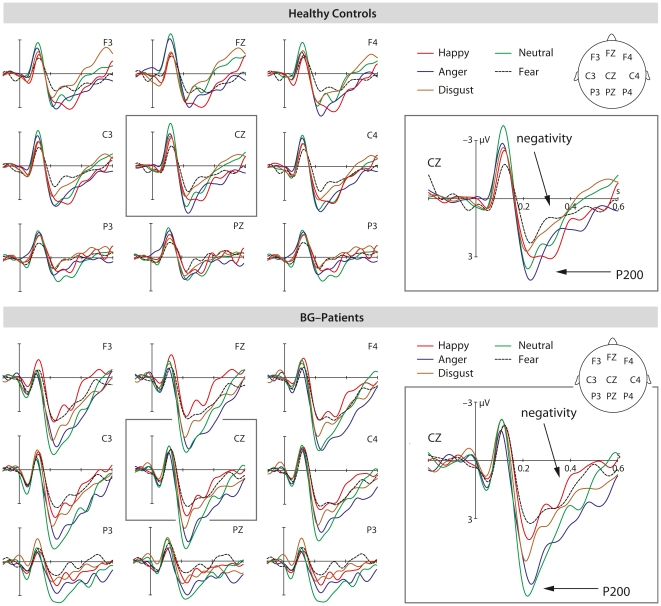
ERP results. The illustration displays the ERP effects at selected electrode-sites elicited by emotional (happy, angry, disgusted, fearful) and neutral sentences for both healthy controls and BG patients.

Overall, the ERP-results confirm comparable emotional cue selection (P200) followed by more elaborate emotional speech processing (negativity-response, see discussion) in BG-patients and healthy controls. Results also suggest faster onset of elaborate processing stages for emotional in contrast to neutral stimuli as reflected in shorter peak-to-peak latencies for emotional stimuli. ERPs are illustrated in Figure 3.

**Figure 4:**
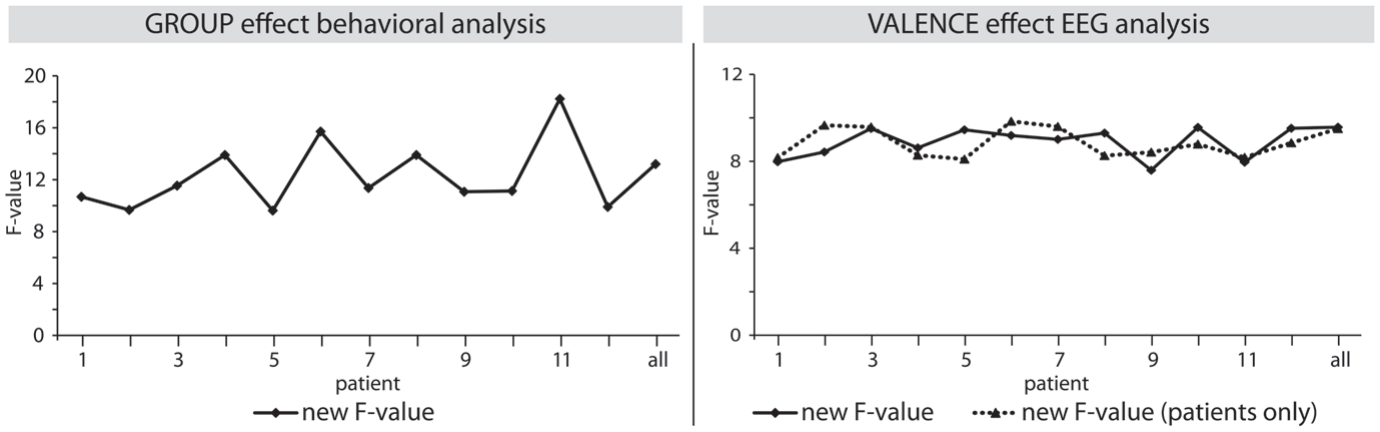
Consistency of Effects. Figure 4a (top) illustrates the consistency of the significant ERP valence effect and the significant group effect (bottom) of the behavioral analysis. Omitted patients (and their respective controls) are listed on the x-axis, the respectively new observed F-value can be seen on the y-axis. The F-value for ‘all’ shows the value obtained when no participant is excluded for comparison reasons.

Finally, a jackknifing procedure (see [42], [46] for a similar approach) was applied to verify that results were not driven by individual patients. If this were the case, ERP and behavioral statistical effects would drop/increase significantly when a single patient is omitted from the statistical analysis. Results from this procedure confirmed the homogeneity of the patient group showing that the reported effects were not driven by individual patients. Figures 4a & b display the consistency of these results.

#### Correlation with neuropsychological test results

To determine whether emotion recognition abilities or the P200 amplitude modulation in patients were related to neuropsychological test scores, a global measure of the patients' accuracy scores and the P200 response was correlated with standardized neuropsychological test scores. Pearson correlations computed among these factors (digit span [forward, backward], alertness [tonic, phasic, divided]) did not confirm a significant correlation between neuropsychological test performance and emotion recognition accuracy and/or observed P200 amplitudes. Correlation matrices are displayed in Table 6.

**Table 6:** Correlations.

Test	Emotional Recognition		P200	
	Pearson Correlation	pvalue	Pearson Correlation	pvalue
DS1	.043	.905	−.519	.125
DS2	−.012	.974	−.434	.210
TAP1	−.609	.062	.058	.873
TAP2	−.546	.103	.196	.587
TAP3	.103	.776	.313	.379
Emotional Recognition	NA		.354	.315
P200	.354	.315	NA	

Comparisons of global measure of patients' emotion recognition accuracy and P200 amplitudes with neuropsychological test scores. Note: DS1 (digit span forward), DS2 (backward), TAP1 (tonic alertness), TAP2 (phasic alertness), TAP3 (divided alertness). NA =  not applicable.

## Discussion

The present study aimed to further specify the role of the BG in emotional speech processing. In particular, we investigated whether the BG are implied during early or late emotional speech processing stages. Late processing stages were explored under implicit and explicit task instructions. The present results revealed that patients suffering from focal lesions in the left BG and healthy controls show comparable ERP responses for early emotional salience detection (P200 component) and the subsequent “emotional representation build up” (negativity). In contrast, we found that healthy controls outperformed patients in a behavioral emotional prosody recognition task (84% vs. 59% correct). No emotion-specific deficit for patients was found, implying a more general emotional prosody recognition deficit in BG patients. However, it can be noted that visual comparison of error patterns revealed that patients categorized neutral sentences as disgust sentences more often than controls. Taken together, the present results suggest that early emotional speech processing is not impaired in patients while later processing is when task instructions are explicit. The dissociation between on-line emotional speech processing and explicit emotional prosody categorization is comparable to evidence obtained from PD patients for different stages of emotional picture processing [47]. We will address the implications for each processing stage in the following paragraphs.

### P200

Different P200 amplitude modulations in response to neutral and emotional speech material replicate previous results from participants of different age groups and have been functionally linked to initial implicit emotional stimulus evaluation [14], [42], [48]. Specifically, we have suggested that the varying P200 amplitude reflects emotional salience detection based on the integration of emotionally significant acoustic cues, that is the neuronal response differentiation is probably based on specific configuration patterns of salient acoustic features (e.g. pitch, voice quality, loudness) signaling the emotional importance of a stimulus [14]. It remains an open question which acoustic parameter predominantly drives this early differentiation (if any single one, see [14]). It is also a matter of debate whether an emotional category can be determined this early, although preliminary evidence points to this possibility [17]. Given the comparable ERPs responses in controls and patients, we conclude that early implicit perceptual emotional differentiation does not critically involve the left BG. This is in line with results from Wieser and colleagues [47] who investigated emotional picture processing in PD patients. The authors also report dissociation between early ERPs (early posterior negativity) and later explicit emotional arousal ratings. Moreover, Schirmer [49] stated that it is commonly found that “low-level prosodic perception” is found unimpaired in patients suffering from BG dysfunction, while later stages that require mapping specific prosodic features such as speech tempo onto emotional representations is found to be impaired. The author argues that a deficiency in speech tempo perception may in turn lead to lower recognition of emotional speech that is strongly signaled through varying tempo/changes. However, results are in contrast to findings reported by [37] who reported a reduced mismatch negativity amplitude in response to sad (but not happy) prosodic deviants, suggesting impairment of pre-attentive emotional prosody processing in PD patients at least for sad stimuli. Two points need to be critically noted with regard to latter finding. First, PD is a neuro-degenerative disease that can lead to functional deficits which are not directly tied to the BG. Observed impairments could thus be related to brain structures (e.g. frontal cortex) that are not affected in the current patient sample. Second, since happy deviants elicited comparable MMNs between healthy controls and PD patients it can be safely concluded that pre-attentive emotional prosody processing is not *generally* impaired in subcortical patients. In the current investigation we did not test sad stimuli. However, as discussed above emotional speech varies as a function of speech tempo [49]. As sadness is an emotion that is usually marked by slower speech rate, the BG may be most sensitive to slowed down speech (see also [50] for a review).

Finally, the idea that the left BG do not play a mandatory role during early, implicit emotional speech processing is reinforced by recent data from our labs. Previously, we have reported unimpaired processing of emotional salient acoustic cues with different stimulus material in the same patient group [7]. In particular, emotional prosodic expectancy violations elicited a comparable positive ERP component (prosodic expectancy positivity, PEP) for healthy controls and patients. Taken together, the present results suggest that the left basal ganglia are not critically involved in early emotional salience detection during implicit emotional task instructions.

### Negativity

Earlier we suggested that initial emotional salience evaluation is followed by a build-up of an emotional representation. That is, individual sentence constituents need to be combined to lead to emotional sentence comprehension. Based on previous results, which suggest at least partially different processing streams for emotional prosody and emotional semantics [9], [10], it can be hypothesized that emotional speech processing requires a continuous combinatorial analysis of emotional features (e.g. emotional prosodic cues, arousal cues, word meaning, etc.). A working model [1] predicts that a first in-depth meaning-related analysis takes place around 400 ms after sentence onset, though it should be noted that earlier meaning-related processing of emotional vocal expressions [18], [10] and visually presented emotional words [51], [52] has been reported. Here, we concentrated on the component that immediately followed the well-described P200. This negativity reached its maximum peak around 300 ms after stimulus onset and peak-to-peak latency was faster for emotional in contrast to neutral sentences, an effect especially pronounced at right frontal electrode sites. This suggests preferential processing of emotional sentences over neutral sentences (see e.g. [38], [53], [54] for rapid & effective processing of emotional information), an effect found for both BG patients and healthy controls. In addition, we report differentiation between neutral and emotional sentences as reflected in enhanced mean amplitudes of this negativity for neutral sentences. We suggest that enhanced amplitudes for emotional sentences may reflect amplified meaning related analysis for these sentences. While evidence for amplified and preferential processing of emotional auditory stimuli is still rare [53], [54], several studies suggest such an advantage for emotional visual stimuli [55]–[60]. For instance, Kissler and colleagues [60] investigated ERPs in response to reading emotional nouns. The authors report an enhanced posterior negativity for emotionally arousing words when compared to neutral words. They attributed the enhanced negativity to preferential processing of emotional words. In particular, they suggest that “emotion acts as a non-valence specific alerting system that enhances initial semantic analysis” ([60], pg. 6). Similarly, Scott and colleagues [51] report enhanced posterior negativities to emotional in contrast to neutral words and suggest preferential processing of emotional words is due to more salient and stronger lexical representations of emotional in contrast to neutral words. Here, we extend the notion of preferential and faster processing of emotional language to the auditory modality in which sentences are emotionally intoned. Based on the observation that our sentences all started with “neutral” words (He has/She has), we can conclude that preferential processing does not only occur for emotional content words, but can also be applied to words that carry no specific emotional meaning but that receive their emotional connotation through the tone of voice that they are uttered in. Interestingly, we do not find differences between BG patients and healthy controls during this processing step, again implying a minor (if any) role of the BG during implicit emotional speech processing and supporting the idea that the BG may only be recruited in tasks or processes which *enforce* an explicit integrative emotional information evaluation [7], [8]. Specifically, dissociation between ERPs and behavioral recognition rates for healthy controls and patients point to the possibility that the BG only come to play a mandatory role when emotional significance and possibly emotional category for a speech stimulus is determined in order to initiate relevant and suitable behavior (see similar idea put forward by [4]).

### Behavioral recognition task

In line with previous findings (e.g., [4], [7], [24], [27]–[30], we report impaired emotional speech recognition in BG patients when compared to healthy controls in an explicit emotional prosody categorization task. This once more suggests that processes that emphasize explicit evaluation and require specific output behavior are particularly impaired in left BG patients. The role of the BG during explicit identification was recently confirmed in an fMRI study [33]. Specifically, the authors suggest that the BG play a dominant role in emotional prosody processing when task instructions enforce explicit processing of the stimulus. This proposal is also in line with the suggestions that cortico-striatal circuits (e.g. projections from frontal cortex to BG and back to cortex via the thalamus) are crucially linked to processing goal-directed behavior [61].

Within the relevant literature, emotion-specific deficits for patients with BG impairments have been reported, especially for stimuli conveying disgust [4], [62]–[65], suggesting that the BG may be particularly involved in the perception of disgust. Here, no emotion-specific deficit was confirmed, but both groups performed less accurately in categorizing disgust sentences. Interestingly, while misclassification or error patterns for emotional speech stimuli were broadly comparable between the two groups, it was also apparent that BG patients misclassified neutral sentences as disgust sentences more often than healthy controls. This could point to a specific role of the BG in disgust processing but given the lack of statistical significance this cannot be confirmed in the current results.

Building on the observation that error patterns were rather similar across groups, it can be hypothesized that patients and controls rely on similar emotional features (acoustic cues, content words) and do not use this information differently, a finding that is in line with the comparable ERP responses in both groups. Thus, it seems as if patients and controls follow similar processing steps (functionally and temporally). We suggest that early stages that require predominantly acoustical feature analyses as well as early more in-depth meaning-related processes do not necessarily recruit the left BG under implicit task instructions. However, as argued previously [8], the BG may be crucial in processes which impose an (integrative) assessment of emotional information, i.e. processes which may rely on sequencing and binding auditory emotional information. Specifically, as mentioned above, emotional speech categorization and recognition deficiencies have been linked to problems in speech tempo perception [49]. Here, speech rate was comparable across emotional categories (except for fear, c.f. Table 3) which may explain why an emotion-specific problem in BG patients can not be confirmed in the present data set. Still, general difficulties in adequately sequencing and extracting temporal information embedded in speech may lead to general recognition problems. In fact, the critical role of the BG within one of the neural timing circuits in mammals was highlighted in a review paper by [66]. The authors proposed that the BG may be involved in a “cognitively controlled timing system that requires attention” ([66], p. 758). While our results cannot directly inform about the interaction between attention and timing per se, we suggest that explicit but not implicit evaluation of emotional speech requires enhanced attention to different cues conveying emotionality (e.g. timing). Future studies should thus directly compare implicit and explicit processing mechanisms in early and late processing stages to support such claims.

### Conclusion

The present investigation is a rare study exploring different processing stages of emotional speech processing in BG patients and healthy controls. Our findings suggest that the BG are not critically involved in all stages of emotional speech processing [7] but specifically underline that it is crucial to distinguish between early rapid and late, more evaluative emotional speech processing stages as evidenced in the dissociation between on-line and off-line processes. In particular, results suggest that BG patients not only follow similar processing steps as healthy controls, but that patients do not suffer from early rapid emotional speech analysis difficulties as reflected in comparable P200 and subsequent negativity ERP amplitudes. Instead, patients with BG lesions perform significantly worse than healthy controls in explicit rating of emotional speech. Taken together, the results suggest specific impairment of executive emotional functions (e.g. decision-making, labeling) in BG patients, implying a role of the BG during late, explicit emotional speech processing stages. Future studies can build on these findings and should explore whether the BG can be implied during rapid and early emotional speech processing when task instructions focus on explicit evaluation of emotional speech.
